# Ensemble Techniques for Robust Fake News Detection: Integrating Transformers, Natural Language Processing, and Machine Learning

**DOI:** 10.3390/s24186062

**Published:** 2024-09-19

**Authors:** Mohammed Al-alshaqi, Danda B. Rawat, Chunmei Liu

**Affiliations:** Department of Electrical Engineering and Computer Science, Howard University, Washington, DC 20059, USA; danda.rawat@howard.edu (D.B.R.); chunmei@howard.edu (C.L.)

**Keywords:** fake news detection, NLP, CNN, multi-modal data, machine learning, BERT, transformers

## Abstract

The proliferation of fake news across multiple modalities has emerged as a critical challenge in the modern information landscape, necessitating advanced detection methods. This study proposes a comprehensive framework for fake news detection integrating text, images, and videos using machine learning and deep learning techniques. The research employs a dual-phased methodology, first analyzing textual data using various classifiers, then developing a multimodal approach combining BERT for text analysis and a modified CNN for visual data. Experiments on the ISOT fake news dataset and MediaEval 2016 image verification corpus demonstrate the effectiveness of the proposed models. For textual data, the Random Forest classifier achieved 99% accuracy, outperforming other algorithms. The multimodal approach showed superior performance compared to baseline models, with a 3.1% accuracy improvement over existing multimodal techniques. This research contributes to the ongoing efforts to combat misinformation by providing a robust, adaptable framework for detecting fake news across different media formats, addressing the complexities of modern information dissemination and manipulation.

## 1. Introduction

The widespread challenge of detecting misinformation, particularly in the form of fake news, has emerged as an essential research priority in modern information distribution. Social media platforms and digital communication channels have heralded an era in which misleading narratives spread easily across several modalities, including text, photos, videos, and speech [1]. To meet this need, modern machine learning and deep learning algorithms play a critical role in ensuring the integrity of information sources. The significance of multimodal false news detection approaches extends beyond theoretical notions, with substantial societal consequences [2]. The consequences of false narratives are diverse and deep in this era of the rapid dissemination of information.

The concept of “fake news” is not a new phenomenon; it has deep roots in society and has risen to the level of a significant problem requiring attention from the research community [3,4]. Recently, the term has evolved, diverting from prior definitions that embraced a wide range of content, encompassing satires, scams, propaganda, and clickbait [5]. Understanding the causes of the spread of fake news is critical to resolving this global challenge. One key factor is the viewers’ lack of information about the source legitimacy and news authenticity [6]. This information void exposes the public to potentially dangerous misinformation. Another factor is the lack of effective automated fact-checking systems [7]. While systems show progress in detecting false news, the manual aspect of their methods makes them time-consuming and incapable of preventing the rapid spread of fake news [8]. Furthermore, multi-modal data, which might come from textual articles, videos, images, and speech, demand a complex analytical approach. When confronted with modified images and sophisticated false narratives, conventional techniques of news verification fail. As a result, deep learning techniques, such as natural language processing (NLP) for text analysis and computer vision for image and video authentication, appear to be a viable alternative [9]. The shortcomings in multi-modal data sets highlight the need for reliable methods for identifying them. Images and videos, which can be easily changed using video editing applications, can distort the facts and spread false narratives. Textual articles, despite their simplicity, might include complex nuances and language manipulations that challenge established verification methods. Speech data add complexity, with artificial voices possibly intensifying false messaging.

In response to these challenges, adopting machine learning models becomes critical, providing an advanced method of detecting detailed patterns and correlations within massive datasets. Spurious patterns in textual content, inappropriate visual features in photos and videos, and anomalies in voice patterns can all be recognized and flagged by combining machine learning techniques. Deep learning models, which use neural networks to imitate human-like learning and decision-making processes, improve detection across multi-modal data sources. Navigating the complexities of the age of technology requires developing and deploying cutting-edge machine learning and deep learning approaches for detecting fake news. These approaches not only deter the concealed transmission of misinformation but also highlight the expanding role of technology in ensuring the credibility of worldwide information dissemination [10].

The framework integrates text, images, and videos to thoroughly detect fake news. We utilize advanced machine learning (ML) and deep learning (DL) methodologies, specifically NLP for text and computer vision for images and videos. A detailed comparison between simpler textual data analysis using traditional machine learning algorithms and complex multi-modal data analysis using deep learning models demonstrates the superior capabilities of the proposed model. We apply BERT (Bidirectional Encoder Representations from Transformers) to integrate textual and visual data and combine BERT with sophisticated deep learning layers to enhance the detection capabilities. Performance assessments indicate the superior accuracy, recall capabilities, and F1-score of the suggested model. These data also suggest the effectiveness of the random forest model in unimodal textual data classification, achieving a 99% accuracy rate. We identifiy and address the specific challenges posed by text, images, videos, and speech data. This paper proposes robust solutions to detect and mitigate the spread of false narratives across these modalities.

In summary, our contributions are as follows:

1. The development of a framework that integrates text, images, and videos for comprehensive fake news detection, leveraging advanced machine learning and deep learning methodologies.
2. The application of BERT to integrate textual and visual data, combining it with sophisticated deep learning layers for improved detection accuracy.
3. The demonstration of the effectiveness of machine learning models in unimodal textual data classification, achieving a 99% accuracy rate. This shows the importance of machine learning models in terms of their complexity for unimodal data.
4. The identification of and attention to the specific challenges posed by text, images, and video data in the context of fake news detection.

Furthermore, the paper is organized into the following sections:

The literature review (Section 2) provides insights into the historical context and definitions of fake news, examines the causes of its spread, and discusses the current state of research in multi-modal data analysis and machine learning approaches for detecting fake news. The methodology (Section 3) details the proposed framework for detecting fake news using multi-modal data, including data collection, preprocessing steps, application of ML and DL techniques, and metrics used for performance evaluation. The results (Section 4) presents the performance outcomes of the proposed model, including a comparison with baseline models. This section highlights the effectiveness of the framework in handling both unimodal and multi-modal data. The conclusions (Section 5) present the most consequential findings and impacts of the study.

## 2. Literature Review

The authors of [11] proposed a unique approach to detect false news by integrating text and photos using a cultural algorithm that also utilizes data gained from situational and normative knowledge. Their model includes multiple components: a sentiment analysis-based textual feature extractor, a visual feature extractor, and a classifier-based false information detector. Extensive trials on real-world multi-modal datasets, such as Weibo and X (formerly Twitter), showed that their method outperformed state-of-the-art algorithms by 9% on average. Singh, Ghosh, and Sonagara [12] presented a multi-modal technique combining text and visual analytics for automated fake news identification. Using the Kaggle Fake News Dataset, their approach involves training classifiers on balanced subsets of fake and credible news articles across 100 iterations. They implemented numerous MLMs, including random forest, logistic regression, and SVM, achieving robust classifier performance through 10-fold cross validation.

Ying et al. [13] introduced the Multi-level pre-trainedodal Cross-attention Network (MMCN) to tackle the challenges of detecting fake news in the mobile internet era. MMCN leverages pre-trained BERT and ResNet models to generate high-quality approximations for text and image features, combined through a multi-modal cross-attention network. Their experiments on the WEIBO and PHEME datasets demonstrated the MMCN’s superior performance over existing models.

Song et al. [14] developed the Cross-modal Attention Residual Network (CARN) and Multichannel Convolutional Neural Network (MCN) within their Cross-modal Attention Residual framework (CARMN). This approach effectively extracts and fuses essential data from different modalities while mitigating noisy information. Their model outperformed state-of-the-art methods in extensive tests across four real-world datasets.

Chen et al. [15] proposed CAFE (Cross-modal Ambiguity-aware Fake News Detection), encompassing a fusion, cross-modal alignment, and ambiguity learning modules. This method adjusts its approach based on cross-modal ambiguity levels, and significantly improves fake news detection accuracy on Twitter and Weibo datasets.

Qian et al. [16] presented the Hierarchical pre-trained Multi-modal Contextual Attention Network (HMCAN), which employs ResNet and BERT for image and text representations, respectively. Their network considers both inter-modality and intra-modality interactions, with hierarchical encoding to capture extensive hierarchical semantics. The HMCAN showed effectiveness across the WEIBO, TWITTER, and PHEME datasets.

Raj and Meel [17] explored multi-modal online information credibility assessments using deep networks such as CNNs and RNNs. Their pre-trained Multi-modal Coupled ConvNet architecture effectively classified online news based on textual and visual information, demonstrating high accuracy across datasets like the TI-CNN, EMERGENT, and MICC-F220.

Choi and Ko [18] focused on detecting misleading videos by combining domain knowledge with multi-modal data fusion. By incorporating domain-specific information and using a linear combination of features, their approach improved detection performance, achieving a 3% gain in accuracy across the test datasets.

Chen, Chu, and Subbalakshmi [19] addressed COVID-19 misinformation with a novel multi-modal dataset and proposed a framework for classifying news as true or false. Their F-Score of this method was estimated at 0.919, and it also achieved 0.882 accuracy in identifying misleading information.

Xue et al. [20] introduced the Multi-modal Consistency Neural Network (MCNN) to detect fake news by extracting and fusing textual and visual features. Their approach demonstrated significant accuracy improvements on several datasets by effectively handling multi-modal data.

Danlei Chen et al. [21] introduced the relevance classifier method and integrated it into the multi-modal framework, with image-text similarity visualization using feature extraction.

Qi et al. [22] identified key textual–image relationships in multi-modal fake news and proposed an entity-enhanced multi-modal fusion method. Their model, which captures critical text-image correlations, was superior in detecting multi-modal fake news.

Singhal et al. [23] developed SpotFake, a multi-modal framework for detecting fake news without relying on extra subtasks, using BERT for text and VGG-19 for image features. When used on datasets obtained from X (formerly Twitter) and Weibo, the algorithm outperformed existing algorithms by an average of 3.27% on X (formerly Twitter) and 6.83% on Weibo. Table 1 shows the most closely related papers with the necessary parameters from the literature.

## 3. Methodology

This section outlines the dual-phased methodology of the research. The main aim of this methodology is to present a separate model for fake news detection based on the nature and dimensions of data. This methodology is not only helpful in identifying more accurate algorithms in terms of accuracy, but it also provides insight into the performance of the algorithms, which leads to better utilization of resources and efficiently identifying false news.

### 3.1. Datasets

The primary ISOT fake news dataset [24] contains textual data from various sources, including political statements, news articles, and press reports from world seminars. It comprises over 40,000 text articles, evenly balanced between true and false classes. The second dataset analyzed is an evolving collection of images shared on social media, notably Twitter, and is available on GitHub [25]. This free corpus evaluates online image verification techniques by leveraging user characteristics and tweeted text. It includes three essential files, serving as a comprehensive resource for confirmed fake and real images Table 2.

The set_images.txt file details the image_id, image_url, annotation (indicating the image’s legitimacy), and associated events. The tweets_images.txt file links each image_id with the tweet’s validity, the event’s origin, and the accompanying tweets. The tweets_images_update.txt file focuses on misleading tweets, specifically those lacking humor or containing false remarks, thereby improving the dataset by retaining tweets with erroneous information. The tweets_event.txt file filters out fabricated tweets that have been deleted or whose accounts have been deactivated. Researchers can use these files in conjunction with set_images.txt to maximize the dataset’s utility.

**Figure 1:**
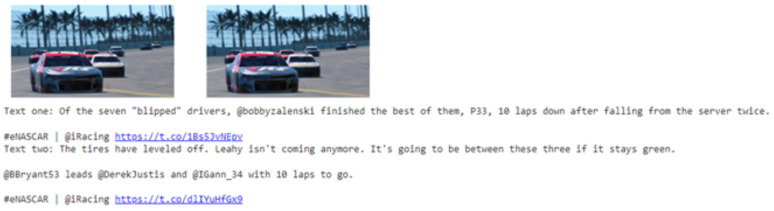
Example images data from MediaEval 2016 dataset.

**Figure 2:**
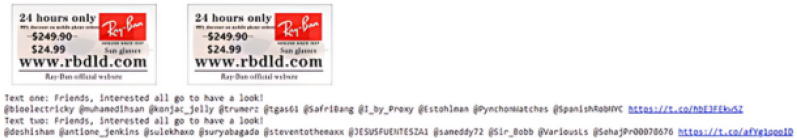
Samples from a multi-modal dataset.

This resource is crucial for computational verification endeavors, offering a fundamental framework for researchers in the field. In addition to features based on user and tweet attributes and forensic features for related images, the dev set and test set files provide Twitter data for training and testing, respectively. This large-scale dataset and its well-structured arrangement support numerous research projects related to social media analysis and computational verification. Figure 1 and Figure 2 illustrate examples images from the MediaEval 2016 dataset.

### 3.2. Proposed Models on Textual Data

The work commenced with meticulous preparation of the textual data using the spaCy natural language processing toolset. The method involved tokenization and cleaning to prepare the text for examination. Afterward, the TF-IDF vectorizer was utilized to transform the preprocessed text data into a numerical representation, establishing the basis for subsequent analysis. To evaluate the chosen classifiers, such as random forest, multinomial naïve Bayes, support vector machine, logistic regression, and k-nearest neighbors, the dataset was carefully split into separate training and testing sets. After receiving training on the specified sets, the accuracy of each classifier was assessed using the testing set. The documentation of the outcomes facilitated the development of a thorough comparison summary table that demonstrates the relative performance of each classifier.

**Figure 3:**
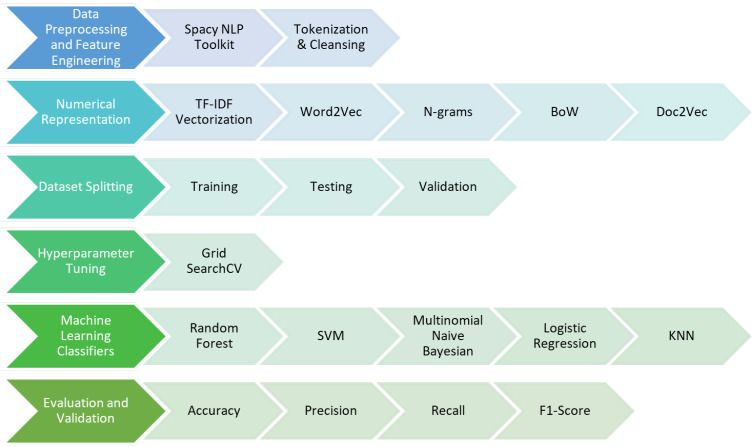
Stepwise architecture of unimodal methodology.

In the case of the random forest classifier, an extra stage was performed to optimize the hyperparameters using Grid Search. The objective of this approach was to enhance the performance of the classifier by identifying the most efficient hyperparameters. After being established, the optimized random forest classifier was trained and evaluated on the testing set. A comprehensive classification report was generated, which provided information on the accuracy, recall, and F1-score. To investigate possible improvements in the performance of the model, different methods of representing features were analyzed. The techniques utilized were TF-IDF, Word2Vec, N-grams, FastText, Doc2Vec, Bag of Words (BoW), and Hashing Vectorizer. The project sought to evaluate the influence of various feature extraction strategies on the overall effectiveness of the models. This methodological framework is thorough and follows a methodical and step-by-step plan. It starts with preparing the data and evaluating the classifier. Then, it moves on to modifying the hyperparameters and finally explores several techniques for representing features. The systematic approach of these processes enhances the rigorous and comprehensive research into the detection of false information within the dataset. Figure 3 represents the architecture diagram of textual data methodology.

### 3.3. Proposed Models on the Multi-Modal Dataset

**Figure 4:**
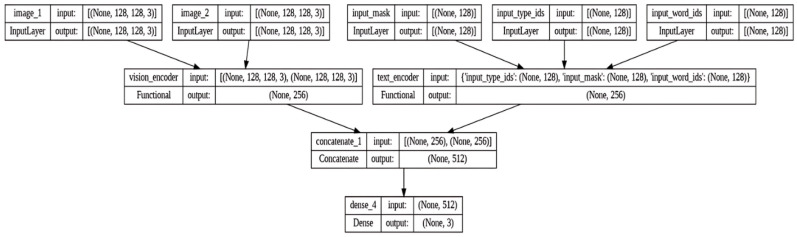
Proposed BERT-based CNN model.

This section presents an improved multi-modal approach that includes a modified Convolutional Neural Network (CNN) structure to accurately identify disinformation. The system consists of several essential elements, including a module that combines multiple features, a feature extractor that incorporates an attention mechanism, a textual feature extractor, and a visual feature extractor. The textual feature extractor begins by carefully preparing the textual data, which includes tokenization, word normalization using metaphor, replacing text-based emojis with sentiment terms, and shortening long sentences. This section presents a sophisticated approach for extracting features from text, utilizing a pre-trained BERT model that is specifically tailored for analyzing Tweet data. The combination of the last four hidden layers of BERT, which are known for their effectiveness in extracting features, produces contextual embeddings. Figure 4 shows the visual encoder used in the multi-modal method Algorithm 1.
**Algorithm 1** Multi-modal Disinformation Detection1:**Input:** Raw text data *T*, Image data *I*2:**Output:** Comprehensive representation C3:**Textual Feature Extraction:**4:   Tokenize text: $T_{t}=\mathrm{tokenize}\left(T\right)$5:   Normalize words: $T_{n}=\mathrm{normalize}\left(T_{t}\right)$6:   Replace emojis: $T_{e}=\mathrm{replace}\_\mathrm{emojis}\left(T_{n}\right)$7:   Shorten sentences: $T_{s}=\mathrm{shorten}\left(T_{e}\right)$8:   Extract BERT embeddings:
$$\;\;\;\;\;\;\;\;\;\;\;E=\mathrm{BERT}\left(T_{s}\right)\;\mathrm{where}\;E=\left[h_{-4},h_{-3},h_{-2},h_{-1}\right]$$9:   Combine embeddings: $T_{f}=\mathrm{combine}\left(E\right)$10:**Visual Feature Extraction:**11:   Pre-trained ResNet V2 model:
$\;\;\;\;\;\;I_{1}=\mathrm{ResNet}\left(I\right)$12:   Fully connected layers:
$\;\;\;\;\;\;I_{f}=\mathrm{FC}\left(I_{1}\right)$13:   Process visual representation:
$\;\;\;\;\;\;I_{m}=\mathrm{process}\left(I_{f}\right)\;\mathrm{where}\;d_{I}=16$14:**Attention Mechanism:**15:   Apply attention:
$$\;\;\;\;\;\;A_{T\to I}=\mathrm{Attention}\left(T_{f},I_{f}\right)$$
$$\;\;\;\;\;\;A_{I\to T}=\mathrm{Attention}\left(I_{f},T_{f}\right)$$
$\;\;\;\;\;\;A_{I\to I}=\mathrm{Attention}\left(I_{f},I_{f}\right)$16:   Fully connected layers with normalization:
$$\;\;\;\;\;\;R_{T\to I}=\mathrm{FC}\left(A_{T\to I}\right)+T_{f}$$
$$\;\;\;\;\;\;R_{I\to T}=\mathrm{FC}\left(A_{I\to T}\right)+I_{f}$$
$\;\;\;\;\;\;R_{I\to I}=\mathrm{FC}\left(A_{I\to I}\right)+I_{f}$17:**Final Processing:**18:   Compress and combine features:
$\;\;\;\;\;\;R_{I\to I}^{\prime }=\mathrm{FC}\left(R_{I\to I}\right)$19:   Fully connected layer with 32 neural units:
$$\;\;\;\;\;\;C={\mathrm{FC}}_{32}\left(\left[T_{f},I_{f},R_{T\to I},R_{I\to T},R_{I\to I}^{\prime }\right]\right)$$

The visual feature extraction process utilizes a pre-trained ResNet V2 model with an input size of 128 × 128 × 3. Two fully connected layers are used, with the output of the second-to-last layer reducing the dimension to a vector size of $d_{I}=16$. This vector forms the final visual representation, denoted as $I_{f}$. The output from the third final layer undergoes additional processing and manipulation to generate a visual feature representation with a dimension of $d_{Im}=16$.

**Figure 5:**
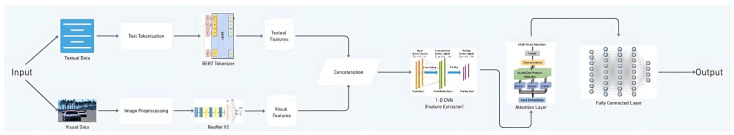
Architecture diagram for multi-modal methodology (proposed model).

The common feature extractor with an attention mechanism presents an enhanced scaled dot-product attention approach $\left(I_{f},I_{m},T_{f},T_{m}\right)$ that may be used for both textual and visual components. This mechanism facilitates the establishment of linkages between the text and images of a post. It incorporates self-attention on images and bidirectional attention processes on text and visual elements. The matrices $A_{tt}^{T\to I}$, $A_{tt}^{I\to T}$, and $A_{tt}^{I\to I}$ are processed using fully connected layers, which incorporate layer normalization and a residual connection. The outcome consists of three vectors: $R_{T\to I}$, $R_{I\to T}$, and $R_{I\to I}$, which represent the combined features. The final step entails compressing the feature vector $R_{I\to I}$ and passing it through a fully connected layer to obtain $R_{I\to I}^{\prime }$. Afterward, a fully connected layer consisting of 32 neural units combines and transmits the outputs $(T_{f},I_{f},R_{T\to I},R_{I\to T},$ and $R_{I\to I}^{\prime })$ to provide a comprehensive representation of both textual and visual features. This improved design seeks to enhance the extraction and integration of textual and visual information to increase the effectiveness of the model in recognizing disinformation. Figure 5 represents the architecture diagram for the proposed methodology.

## 4. Results

### 4.1. Comparison of Machine Leaning Algorithms on a Unimodal Textual Dataset

Different ML algorithms were used to assess various classification models and their performance in identifying fake news, followed by examining their performance. The table presents the essential parameters of these algorithms: F1-scores, accuracy, recall, precision. It also summarizes the results across several modalities. With a 99% accuracy rate and high precision, recall, and F1-score values at 0.99, the random forest classifier proved effective. With a 95% accuracy rate, the support vector machine (SVM) demonstrated impressive performance. Its precision, recall, and F1-score metrics were also good, at 0.96, 0.94, and 0.95, respectively. The k-nearest neighbors (KNN) and logistic regression demonstrated excellent accuracy rates of 92% and 91%, respectively, along with balanced precision, recall, and F1-score values. Multinomial naïve Bayes, while slightly lower in accuracy at 88%, demonstrated consistent precision, recall, and F1-score metrics, at 0.89, 0.87, and 0.88, respectively. These results underscore the effectiveness of the random forest model and the competitive performance of the other classifiers in discerning fake news within the dataset. Table 3 shows the results of the textual dataset.

### 4.2. Result of the Proposed Model on a Multi-Modal Dataset

To assess our suggested model’s effectiveness in the field of false news detection, we carried out a thorough and rigorous comparison study throughout our trials with some baseline unimodal and multi-modal models. TextLSTM was the first LSTM network in the lineup. It was unique in that it had three layers: a bidirectional LSTM, a softmax layer, and a feed-forward layer. Interestingly, this model used 32-dimensional pre-trained word embeddings from Google, concentrating only on textual aspects in its methodology [5]. Textual (BERTweet) [2] was then studied, which uses the pre-trained BERTweet model as the only source of textual features. On the contrary, the visual model focused only on the visual components taken from the VGG-19 architecture. Lastly, Spotfake was a convergence of multi-modal traits that came from VGG-19 and BERT [23]. In comparison to these models, our proposed model for multi-modal data has the capability of textual analysis using BERT and can also analyze Images with the utilization of CNN layers.

**Figure 6:**
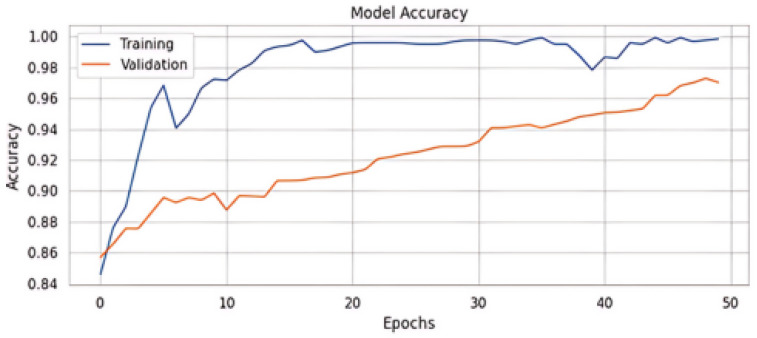
Accuracy of proposed model.

**Figure 7:**
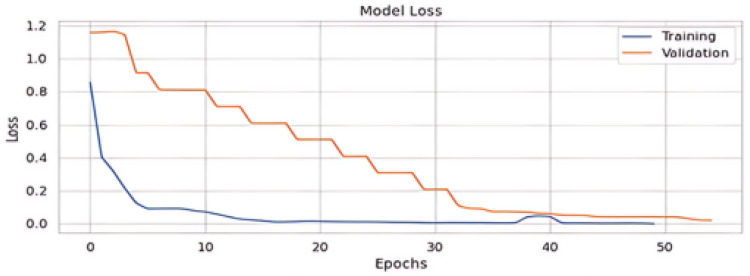
Loss of proposed model.

The results are depicted in Table 4, showing the comparison between our suggested technique and the baseline using the MediaEval 2016 dataset. Interestingly, our suggested model outperformed several multi-modal models and baseline unimodal models. Our model significantly outperformed Spotfake, a model that included multi-modal features, delivering a 3.1% gain in accuracy. Table 4 provides the results of the multi-modal dataset. Table 5 shows the normalized confusion matrix, and Figure 6 shows the accuracy and loss curves of the proposed method Figure 7.

## 5. Conclusions

This study confronts the intricate challenge of misinformation detection, with a particular focus on fabricated news in today’s digital age. By advocating for a robust, systematic approach leveraging advanced machine learning and deep learning techniques, the research introduces a multi-modal architecture that combines natural language processing (NLP) for text analysis with computer vision for image and video verification. This framework’s capacity to analyze diverse forms of communication—written content, images, and videos—significantly enhances its ability to discern genuine news from misleading information. The model, evaluated using the MediaEval 2016 dataset, demonstrates an improved accuracy, precision, recall, and F1-score, reflecting its effectiveness in tackling contemporary media challenges. Future research could explore evaluating multilingual models to include text data in various languages and developing lightweight models for real-time fake news detection. These advancements could further enhance the practical applications and the framework’s adaptability to diverse linguistic and operational environments.

The study highlights the exceptional performance of the random forest model, achieving a 99% accuracy rate. However, it is essential to consider the model’s limitations, such as its potential overfitting to specific datasets and the computational resources required for deployment. Random forest may not always be the best choice in scenarios involving high-dimensional or sparse data or real-time processing needs, where algorithms like support vector machine or neural networks could perform better. The MediaEval 2016 dataset, while valuable, may not fully represent the diversity and complexity of global misinformation. Future work should incorporate additional datasets to ensure the framework’s robustness across various types of misinformation. Additionally, addressing trade-offs in model choice and evaluating scalability with increasing data volume and complexity is critical for optimizing performance.

The social impact of this research is significant. By improving the detection of fake news, the framework can contribute to increasing social trust and reducing the societal divisions caused by misinformation. Incorporating user feedback into the framework can enhance its usability and effectiveness in real-world settings. Optimizing resource use without compromising performance is crucial, especially for deploying the framework in practical applications. Future research should also explore methods for efficient resource management and strategies for scaling the model effectively. These considerations will help ensure that the framework remains practical and impactful, addressing the global challenge of disinformation comprehensively.

## Figures and Tables

**Table 1:** Summary of fake news detection methodologies.

Ref	Methodology	Datasets Used	Evaluation Metrics
Singhal et al., 2019 [23]	SpotFake framework, BERT, and VGG-19	Twitter, Weibo	Outperformed state-of-the-art models by 3.27% and 6.83%
Shah and Kobti, 2020 [11]	Cultural algorithm for fake news detection; independent detection from text and images	Weibo, Twitter	Accuracy improvement of 9% on average
Singh, Ghosh, and Sonagara, 2021 [12]	Logistic regression, linear discrimination analysis, quadratic discriminant analysis, k-nearest neighbors, naïve Bayes, support vector machine, classification and regression tree, and random forest	Kaggle Fake News Dataset	Various classifiers, 70–30 train/test split
Ying et al., 2021 [13]	MMCN model leveraging BERT and ResNet	Weibo, PHEME	Not specified
Song et al., 2021 [14]	CARMN framework with Crossmodal Attention Residual Network (CARN) and Multichannel CNN (MCN).	Four real-world datasets	Not specified
Raj and Meel, 2021 [17]	Coupled ConvNet architecture; comparative analysis with various CNN models.	TI-CNN, EMERGENT, MICC-F220	High accuracies, outperformed state-of-the-art methods
Chen, Chu, and Subbalakshmi, 2021 [19]	Introduction of the multi-modal dataset on COVID-19 vaccine news; proposal of a fake news detection architecture	COVID-19 vaccine news dataset	F-Score of 0.919, accuracy of 0.882
Qian et al., 2021 [16]	HMCAN model utilizing BERT and ResNet	Weibo, Twitter, PHEME	Outperformed state-of-the-art baselines
Qi et al., 2021 [22]	Entity-enhanced multi-modal fusion framework	Not specified	Outperformed current state of the art
Xue et al., 2021 [20]	MCNN framework with five subnetworks	Four commonly used datasets	Clear improvement in detection accuracy
Chen et al., 2022 [15]	CAFÉ (Cross-modal Ambiguity-aware Fake news detection)	Twitter, Weibo	Accuracy improvement by 2.2–18.9% and 1.7–11.4%
Choi and Ko, 2022 [18]	Integration of domain knowledge and multi-modal data fusion, Linear combination	FVC 2.0, FVC 3.0, Volunteer Annotated Video Dataset, Misleading Youtube Video Corpus	F1-score of 0.93, outperformed the comparison models
Danlei Chen et al., 2023 [21]	Relevance classifier method	Not Specified	Not Specified

**Table 2:** Summary of datasets for fake news and image verification.

Dataset	Content	Size	Balance	Purpose	Files Included
ISOT Fake News Dataset	Textual data from political statements, news, and press reports	Over 40,000 articles	Balanced (true and false articles)	To study and analyze fake news characteristics and patterns	N/A
Image Verification Corpus	Fake and real images shared on social media, notably Twitter	Evolving dataset	N/A	To evaluate online image verification techniques using user and tweet data	’set_images.txt’: image_id, image_url, annotation, events; ’tweets_images.txt’: image_id, tweet validity, event origin, accompanying tweets; ’tweets_images_update.txt’: misleading tweets, erroneous information; ’tweets_event.txt’: deleted or deactivated account tweets

**Table 3:** Results of textual dataset.

Model	Accuracy	Precision	Recall	F1-Score
Random forest	0.99	0.99	0.99	0.99
Support vector machine	0.95	0.96	0.94	0.95
Logistic regression	0.92	0.93	0.91	0.92
Multinomial naïve Bayes	0.88	0.89	0.87	0.88
K-nearest neighbors	0.91	0.92	0.90	0.91

**Table 4:** Results for multi-modal dataset.

Model	Accuracy	Precision	Recall	F1-Score
TextLSTM (de Sarkar, Yang, and Mukherjee, 2018) [5]	0.512	0.576	0.543	0.564
Textual (BERTweet) (Duc Tuan and Quang Nhat Minh, 2021) [2]	0.656	0.657	0.820	0.733
Att-RNN (Huy et al., 2021) [9]	0.654	0.739	0.605	0.667
MVAE (Choi and Ko, 2022) [18]	0.735	0.791	0.709	0.748
Spotfake (Singhal et al., 2019) [23]	0.768	0.741	0.890	0.802
Proposed model	**0.944**	**0.902**	**0.911**	**0.892**

The bold formatting in the table is used to highlight the highest results for Accuracy, Precision, Recall, and F1-score.

**Table 5:** Normalized confusion matrix for proposed model.

	Positive	Neutral	Negative
Positive	0.95	0.02	0.03
Neutral	0.04	0.94	0.02
Negative	0.03	0.03	0.94

## Data Availability

Data are contained within the article.
